# Supplementary material for: In Depth Topological Analysis of Arabidopsis Mid-SUN Proteins and Their Interaction with the Membrane-Bound Transcription Factor MaMYB
Source: Plants (Basel). 2023 Apr 27;12(9):1787. doi: 10.3390/plants12091787 (PMC10180911; doi:10.3390/plants12091787)
Supplement: Supplementary file 1 [file plants-12-01787-s001.zip › plants-2318406-supplementary.pdf]

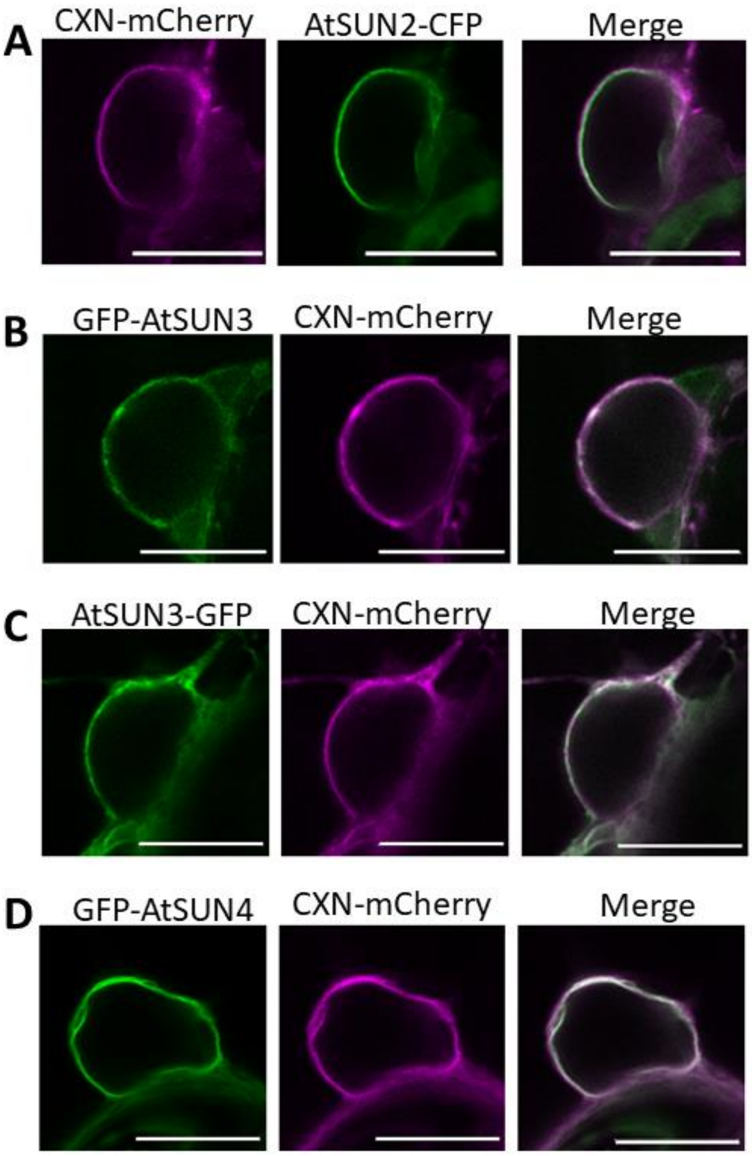

Figure S1: Co-localisation of CXN-mCherry with AtSUN2-CFP, AtSUN3-GFP, GFP-AtSUN3 and GFP-AtSUN4.

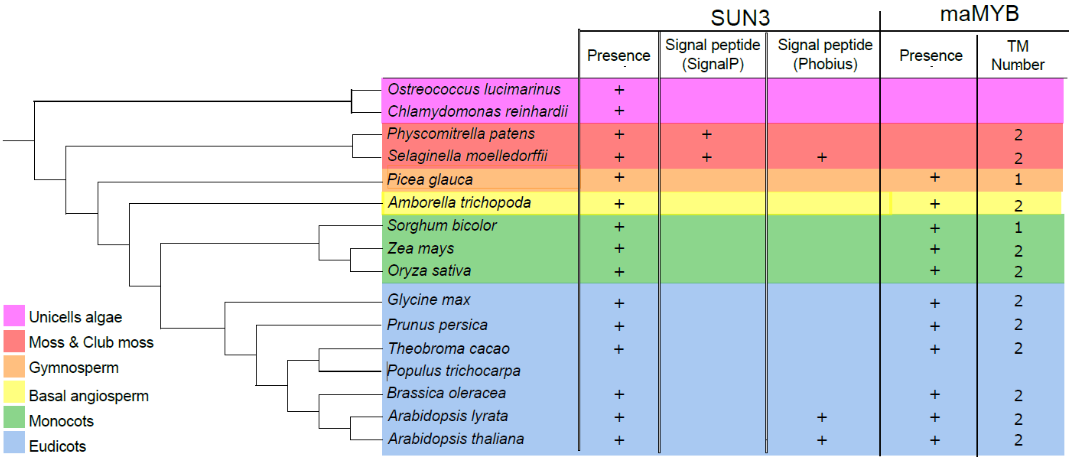

	SUN3			maMYB	
	Presence	Signal peptide (SignalP)	Signal peptide (Phobius)	Presence	TM Number
Ostreococcus lucimarinus	+				
Chlamydomonas reinhardii	+				
Physcomitrella patens	+	+			2
Selaginella moelledorffii	+	+	+		2
Picea glauca	+			+	1
Amborella trichopoda	+			+	2
Sorghum bicolor	+			+	1
Zea mays	+			+	2
Oryza sativa	+			+	2
Glycine max	+			+	2
Prunus persica	+			+	2
Theobroma cacao	+			+	2
Populus trichocarpa					
Brassica oleracea	+			+	2
Arabidopsis lyrata	+		+	+	2
Arabidopsis thaliana	+		+	+	2

Figure S2: SUN3 is present all across the green lineage when maMYB is only conserved in land plants.

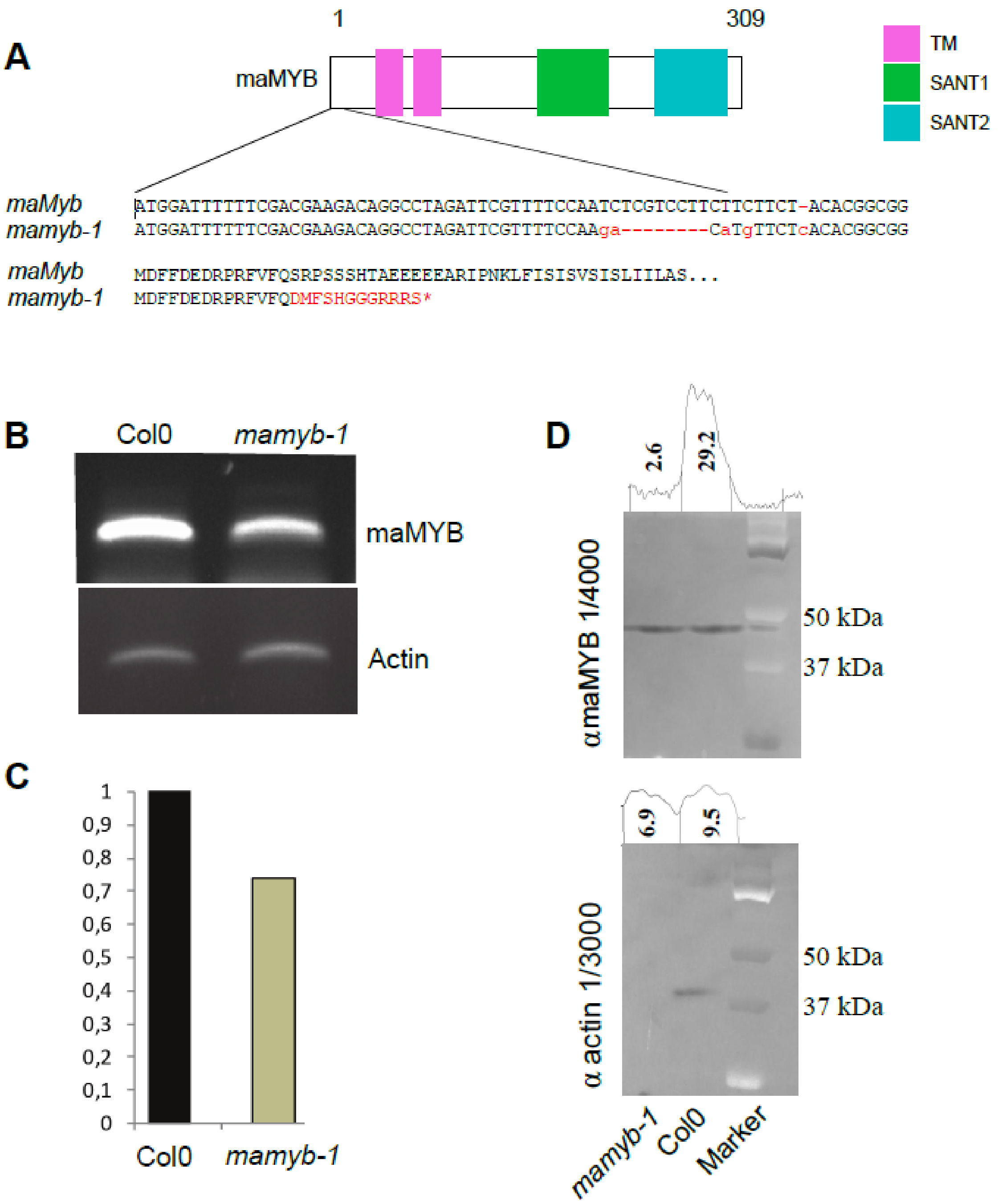

Figure S3: Characterisation of *maMYB-1* mutant generated by CRISPR/Cas9.

Supplementary Table S1: Percentage change in ER structure variables

The percentage change from control ER structure calculated using Tukey HSD after ANOVA comparisons. The significance of the difference from control is shown by * p<0.05; ** p<0.01 and ***p<0.001.

ER subdomain	Variable	Tukey HSD post hoc test p-value and percentage change from control			
		RFP-SUN3	SUN3-RFP	RFP-SUN4	SUN4-RFP
Tubule	Length	12.10%	0.31%	* -16.30%	2.27%
Cisternae	Area	*** 85.30%	*** 245.48%	-21.15%	-32.36%
	Circularity	8.40%	7.21%	9.33%	11.76%
	Elongation	-5.60%	-6.33%	* -10.65%	* -10.35%
	Roughness	-6.50%	1.29%	-17.77%	-22.82%
Polygonal region	Area	20.10%	5.28%	* -11.75%	24.34%
	Circularity	*** 8.06%	** 12.49%	-5.52%	* -7.69%
	Roughness	*** -8.66%	*** -12.52%	5.05%	* 6.93%
	Elongation	* -5.57%	* -7.86%	2.27%	4.24%

Supplementary Table S2. Screen for SUN3 interactors using Membrane Yeast-Two Hybrid (MYTH) system.

Summary of protein interactions using full-length SUN3 as bait

TAIR ID	Protein	Description	Subcellular location
AT2G23310	RER1C1	ER to Golgi retrograde traffic	Golgi membrane
AT2G40620	bZIP18	Transcription facto	Nucleus/Cytoplasm
AT4G32530	VMA15	Transmembrane ATPase	Vacuole
AT5G45420	maMYB	Membrane anchoredTranscription factor	Nucleus/Endoplasmic reticulum

Supplementary Table S3. FRET efficiency (EF) indicates interactions, or lack thereof. Positive protein-protein interactions are displayed in green.

Construct combinations	Experimental E_F ± S.E.M	Control E_F ± S.E.M	p-Value [t-test]
**YFP-*At*SUN1 + *At*maMyb-CFP + p19**	-2.72 ± 1.22	-1.2 ± 0.18	0.23
**YFP-*At*SUN2 + *At*maMyb-CFP + p19**	-0.03 ± 1.11	-1.21 ± 0.13	0.29
**CFP-*At*maMyb + YFP-*At*SUN3 + p19**	**0.64 ± 0.56**	**-0.84 ± 0.13**	**0.01**
***At*maMyb-CFP + YFP-*At*SUN3 + p19**	**0.32 ± 0.41**	**-0.96 ± 0.14**	**0.005**
**CFP-*At*SUN4 + YFP-*At*maMyb + p19**	-1.99 ± 0.98	-0.92 ± 0.27	0.68

Supplementary Table S4: Orthologues of SUN3 and maMYB in 16 plant species. Orthologues were collected from the OMA (green) and EggNOG (blue) databases.

Species	maMYB	SUN3
Arabidopsis thaliana	ARATH37221	ARATH03555
Arabidopsis lyrata	ARALY31851	ARALY02450
Brassica oleracea	BRAOL38657	BRAOL27708
Populus trichocarpa	POPTR28163	POPTR24932
Theobroma cacao	THECC12053	THECC25548
Prunus persica	PRUPE01063	PRUPE03058
Glycine max	SOYBN56837	SOYBN70811
Oryza sativa sub sp indica	ORYSI33124	ORYSI04454
Sorghum bicolor	SORBI33422	SORBI20837
Zea mays	MAIZE02300	MAIZE39878
Amborella trichopoda	AMBTC12349	AMBTC06464
Picea glauca	PICGL22253	PICGL07507
Selaginella moellendorffii	SELML04218	*88036.EFJ30416*
Physcomitrella patens	PHYPA19859	*3218.PP1S191_3V6.1*
Chlamydomonas reinhardtii	absent	*3055.EDP00650*
Ostreococcus lucimarinus	absent	*242159.ABO96635*
